# Strength training intervention with creatine/whey protein supplementation vs placebo on frailty in older adults with cirrhosis (STRIVE): A Randomized Controlled Trial

**DOI:** 10.21203/rs.3.rs-10695023/v1

**Published:** 2026-09-19

**Authors:** Melinda Wang, Dorothea Kent, Kathy Pariani, Jessica Elia, Srilakshmi Seetharaman, Catherine Lee, Julian Mansour, Carly Mak, Puneeta Tandon, Jennifer Lai

**Affiliations:** University of California-San Francisco; University of California-San Francisco; University of California-San Francisco; University of California-San Francisco; University of California-San Francisco; University of California-San Francisco; University of Alberta; University of Alberta; University of Alberta; University of California-San Francisco

**Keywords:** frailty, cirrhosis, protein, creatine, exercise, older adults, randomized controlled trial, placebo

## Abstract

**Background:**

Older adults with cirrhosis are particularly vulnerable to developing frailty with poor outcomes including increased falls, physical disability, hospitalizations, and all-cause mortality. Current management of frailty among older adults with cirrhosis is limited to general lifestyle recommendations including increased exercise and optimized diet, and few studies examining exercise interventions include patients with Child Pugh Class B or C. Whey protein and creatine are well-studied supplements commonly used to improve physical performance in athletes and have recently demonstrated positive effect in muscle mass among older adults but have not been studied in patients with cirrhosis. Therefore, we aimed to evaluate the effect of creatine/whey protein supplementation on frailty in older adults with cirrhosis (Child Pugh Class A, B, C) engaged in Strength Training Intervention (STRIVE), a semi-supervised program practicing exercise, nutrition, and life skills.

**Methods:**

This study is a randomized, placebo-controlled trial including older adults (≥ 60 years) with cirrhosis evaluated at a single health center. We will recruit 200 patients informed by sample size calculations. Patients will be randomized 1:1, stratified by Child Pugh Class and gender. Participants who meet eligibility criteria will be automatically enrolled in a 2-week “ramp-up” period to assess engagement in the exercise portion of STRIVE prior to full enrollment into the 12-week study (STRIVE with creatine/whey protein supplementation vs placebo). We will assess health-related quality of life using Patient Reported Outcomes Measurement Information System (PROMIS-29 v 2.0), self-reported physical activity using Duke Activity Status Index (DASI), patient activation using Patient Activation Measure (PAM^®^), and diet quality by healthy eating index using DietID^®^ and Automated Self-Administered Dietary Assessment Tool (ASA24). Body composition scan (BIA), liver frailty index (LFI), and morphologic measurements including waist circumference, hip circumference, midarm muscle circumference, and triceps skin fold will be measured. The primary outcome will be physical frailty (by LFI); secondary outcomes will include health-related quality of life (HrQoL), physical activity, healthy eating index, and body composition.

**Discussion:**

Our results will help inform frailty-mitigating strategies among older adults with cirrhosis that can be used for patients prior to or on the liver transplant waitlist. The addition of supplementation with creatine/whey protein may add to the armamentarium of strategies to improve frailty within this vulnerable population.

**Trial Registration:**

NCT07029243 (Registration Date: 2025-05-15)

## Background

Frailty, the loss of physiological reserve and increased vulnerability to health stressors, is a common muscle dysfunction-related condition among older adults.^1, 2^ Among community-dwelling non-nursing home adults ≥ 65 years, an estimated 15% of patients are frail and 45% are pre-frail.^2^ Frailty is associated with poor health outcomes including increased hospitalizations, falls, and mortality.^1-5^ Among adults with cirrhosis, frailty occurs in 1 in 4 adults, independent of age, and leads to the development of malnutrition, portal hypertension, impaired ammonia metabolism, and sarcopenia, all of which lead to hepatic decompensation and worse frailty. ^6-13^ Older adults with cirrhosis, particularly with decompensation, are therefore a particularly vulnerable population of patients who develop compounded frailty from age-related and cirrhosis-related factors.

Several strategies have been evaluated to improve sarcopenia and frailty. Exercise and nutrition are mainstay therapies to mitigate frailty and sarcopenia among both older adults and patients with cirrhosis.^14-16^ Nutrition-specific therapies that lead to improved outcomes include nutritional counseling, nocturnal feeding, supplementation with branched-chain amino acids, and increased protein intake.^17, 18^ Exercise-specific interventions associated with improved outcomes include aerobic exercise, moderate exercise, tai-chi, yoga, and strength-training exercises.^18, 19^ Geriatric assessments and medication optimization have also been effective strategies to reduce hospitalizations and further functional decline.^2^ Finally, protein and creatine supplementation have been evaluated among older adults who are frail given their promising effects on exercise tolerance and rehabilitation among athletes.^20-24^ National guidelines recommend prescribing prehabilitation strategies, including protein supplementation and physical therapy, for adults with decompensated cirrhosis who are frail given the particularly high risk of mortality in this patient population.^8^

Creatine is a nitrogenous organic acid found predominantly in skeletal muscle (> 95%) in the form of phosphocreatine and free creatine. About half of the body’s daily requirement of creatine is derived from diet. Its role centers on the conversion between creatine and phosphocreatine which yields free energy in the form of adenosine triphosphate (ATP).^25^ Whey protein is a high concentration of essential amino acids that increase muscle protein synthesis rate particularly after resistance training.^26^ The combination of creatine with resistance training among older adults was associated with greater lean muscle mass strength compared to those with resistance training only.^27^ Similarly, the combination of whey protein and creatine supplementation in addition to resistance lead to improved muscle mass and strength compared to creatine supplementation alone or to placebo.^23^ Protein supplementation, including branched-chain amino acids, is a cornerstone of the management of frailty among patients with cirrhosis.^28^ However, the use of creatine/whey protein supplementation has not been evaluated among older adults with cirrhosis.

In this randomized trial, we aim to evaluate the efficacy of creatine/whey protein supplementation vs placebo, each combined with a semi-guided digital health application, Strength Training Intervention (STRIVE), focused on exercise three days a week, 30 minutes per session, personalized nutrition counseling, and life skills support on frailty among older adults with cirrhosis, including patients with decompensated cirrhosis. We hypothesize that patients undergoing STRIVE with whey protein/creatine supplementation will experience improved frailty compared to those undergoing STRIVE with placebo supplementation.

## Methods/Design

### Study design and setting

**Figure 1:**
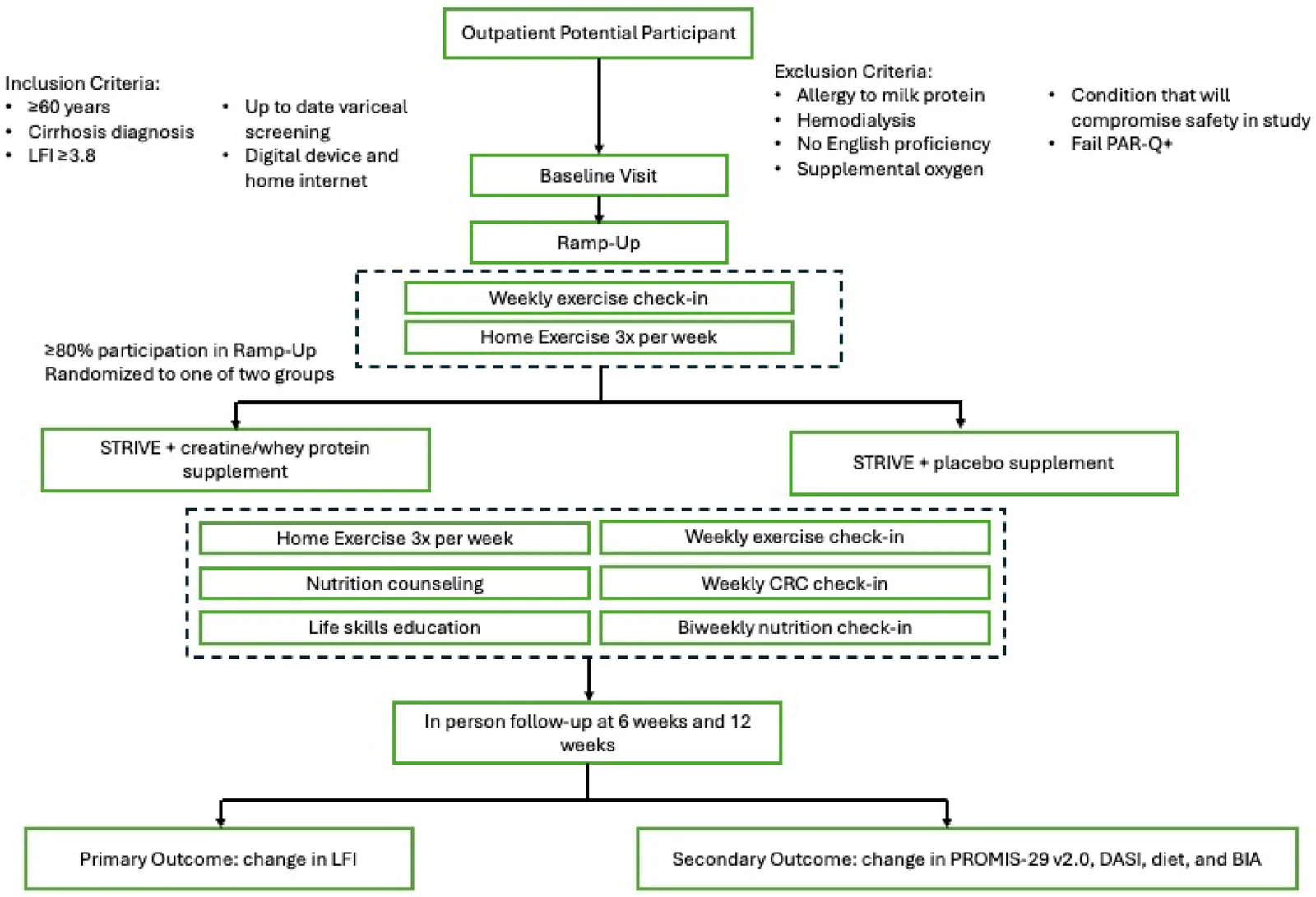
Study Design LFI: liver frailty index, PAR-Q: Physical Activity Readiness Questionnaire for Everyone, PROMIS-29 v2.0: Patient Reported Outcomes Measurement Information System, DASI: Duke Activity Status Index, BIA: Bioimpedance analysis

**Figure 2:**
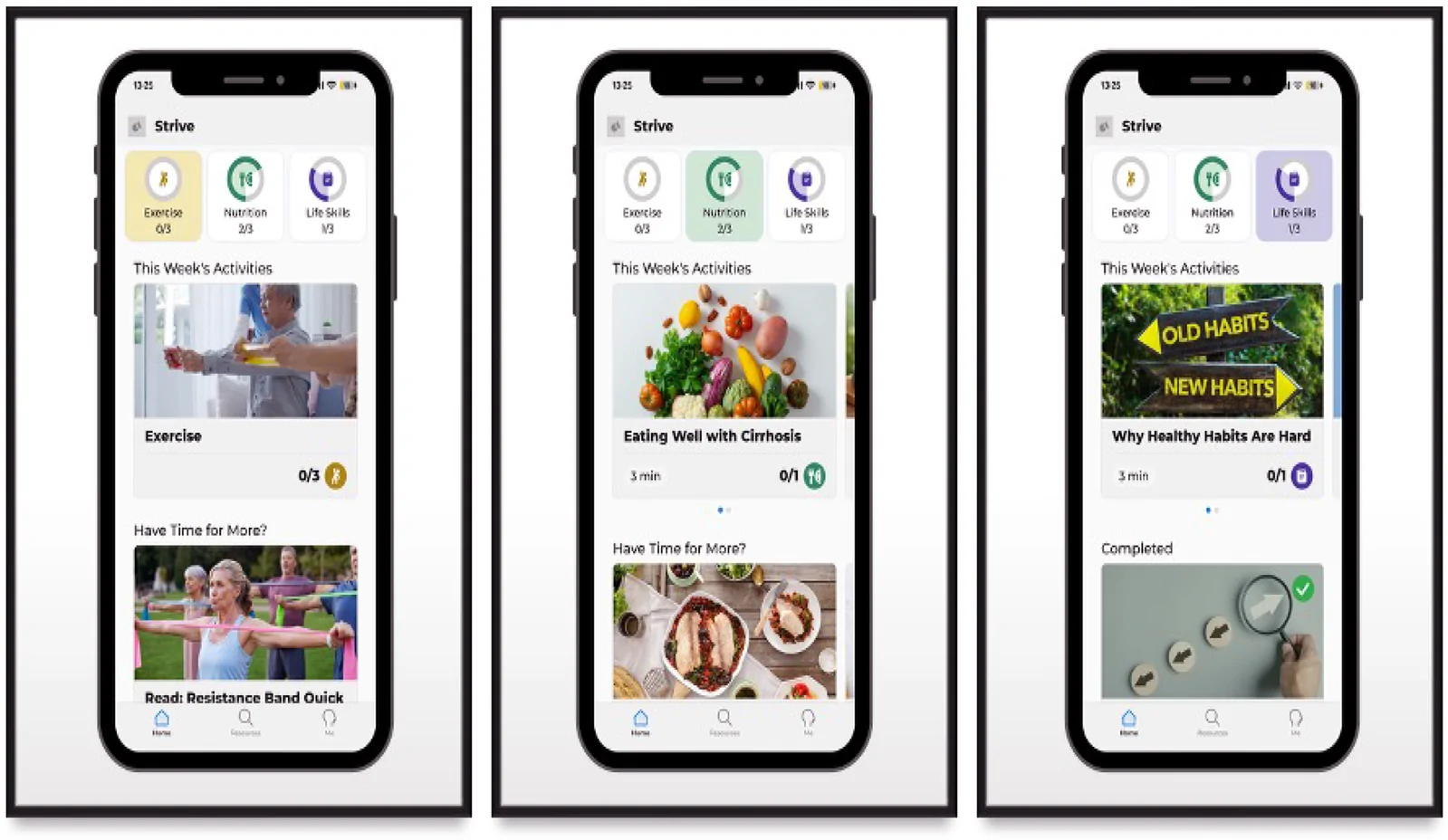
STRIVE Digital Health Application Interface

We will conduct a prospective double-blind placebo-controlled, two-arm randomized controlled trial at a single center (Fig. 1). To ensure patients in the study are able to navigate the STRIVE platform and perform exercises on STRIVE, all eligible patients will first be pre-enrolled in a two-week “ramp-up” period during which they will participate in a simplified version of the digital health program that includes weekly exercise videos, post-video surveys, and weekly telehealth check-ins with a physician and/or exercise specialist to monitor their weekly exercise and adjust exercise prescriptions as needed. Patients who achieve ≥ 80% engagement with app activities and scheduled check-ins during the ramp-up period will proceed to randomization. Patients enrolled in the study will be randomized to creatine/whey protein supplementation vs placebo supplementation and STRIVE digital health programming. The STRIVE digital health programming uses the same interface as the ramp-up but includes three pillars: exercise, nutrition, and life skills (Fig. 2). Activities in the app include pre-recorded exercise videos to be completed three times per week, educational videos and reading, and interactive quizzes and surveys. Patients also receive weekly physician or exercise specialist check-ins to monitor exercise progression and to recommend personalized modifications; weekly check-ins with clinical research coordinator for safety monitoring, app-related questions, and supplement adherence evaluation; and biweekly check-ins with a nutritionist to assess dietary habits and provide personalized dietary recommendations. Patients are evaluated in-person at baseline prior to the ramp-up period, at 6 weeks after enrollment into STRIVE, and at 12 weeks at completion of STRIVE.

LFI: liver frailty index, PAR-Q: Physical Activity Readiness Questionnaire for Everyone, PROMIS-29 v2.0: Patient Reported Outcomes Measurement Information System, DASI: Duke Activity Status Index, BIA: Bioimpedance analysis

### Inclusion Criteria

Inclusion criteria include adults ≥ 60 years old with confirmed diagnosis of cirrhosis (Child Pugh Class A, B, or C) and a liver frailty index (LFI) ≥ 3.8. Participants must also be up to date on variceal screening through either up-to-date endoscopy screening and/or surveillance or on beta blocker. All participants require access to a digital device and internet, ability to remain in the study for at least 3 months, and engagement in ≥ 80% of activities in the ramp-up period before enrollment into STRIVE.

### Exclusion Criteria

Patients who meet the following criteria will be excluded: (1) inability to safely take creatine/whey protein supplementation due to allergy to milk protein, (2) hemodialysis due to concern for possible renal effects of creatine/whey protein supplementation, (3) lack of internet-connected smart device, (4) no English language proficiency due to English interface of STRIVE, (5) on supplemental oxygen due to safe home exercise engagement and possible edema associated with creatine/whey protein supplementation, (6) or with a condition that would compromise the safety of the patient participating in home exercise including unstable angina, myocardial infarction within the past 6 months, uncontrolled arrhythmia, severe COPD, pulmonary embolism within 1 year, bone fracture within 1 year, severe osteoarthritis precluding exercise, or ≥ 2 Grade 2 hepatic encephalopathy. Additionally, patients who fail to meet exercise screening criteria based on the Physical Activity Readiness Questionnaire for Everyone (PAR-Q+)^29^ will be excluded from the study.

### Randomization

Patients who meet eligibility criteria and complete the two-week ramp-up period will be randomized using a 1:1 allocation ratio to placebo vs creatine/whey protein supplementation and permuted blocks of sizes of lengths 2 and 4 by the statistician. Randomization will be stratified by Child Pugh score (A, B, C) and gender (women, men). Six randomization lists will be generated by the study biostatistician using R “randotools” package for each combination of stratification factors: A/female, B/female, C/female, A/male, B/male, C/male. The randomization list will be used to assign eligible and consented participants to one of the two trial arms of the study to the pharmacy. Patients will receive a blinded box of creatine/whey protein vs placebo in single daily sachets with instructions on use.

### Measurements

#### Assessments

**Figure 3:**
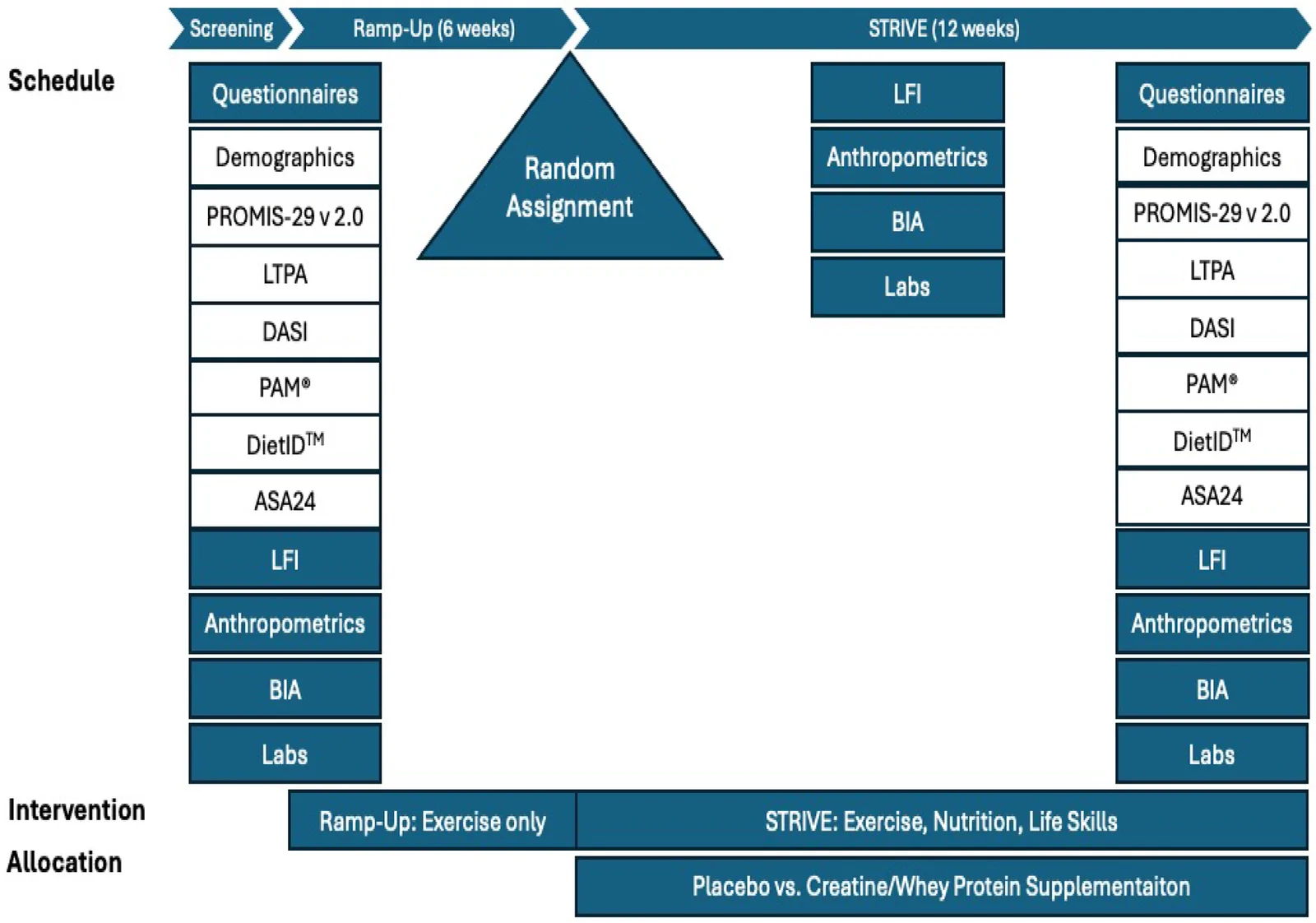
Schedule of Activities LFI: liver frailty index, LTPA: Godin-Shephard Leisure Time Physical Activity Index, PROMIS-29 v2.0: Patient Reported Outcomes Measurement Information System, DASI: Duke Activity Status Index, BIA: Bioimpedance analysis, ASA24: Automated self-administered 24-hour Dietary Assessment, PAM^®^: Patient Activation Measure

Baseline assessment will be completed prior to pre-enrollment into the ramp-up period (Fig. 3). Additional follow-up assessments will be completed at 6 weeks after enrollment into the study and 12 weeks at completion of the study. Assessment at baseline will include demographic characteristics, vital signs and physical exam, and the following questionnaires: Patient Reported Outcomes Measurement Information System (PROMIS-29 v 2.0) to measure health-related quality of life^30^ (HrQoL), Godin-Shephard Leisure Time Physical Activity Index (LTPA)^31^ and Duke Activity Status Index (DASI)^32^ to measure self-reported physical activity, the Patient Activation Measure (PAM^®^)^33^ to measure patient activation, and DietID^™^^34^ and the Automated Self-Administered Dietary Assessment Tool (ASA24)^35^ to measure dietary patterns. PROMIS-29 v2.0 is an NIH-supported HrQoL measure of 29 questions that cover broad domains including physical function, anxiety, depression, fatigue, sleep disturbance, participation in social roles and activities, and pain intensity with built-in normative references to the US general population.^30^ The LTPA is a 3-question survey that measures how much strenuous, moderate, and mild exercise a person completes in a 7-day period. A weekly leisure-time activity score is calculated to categorize a patient’s activity level.^31^ The DASI is a validated 12-question survey designed to estimate a patient’s functional capacity.^32^ The Patient Activation Measure is a 13-item survey to measure a patient’s knowledge, skill and confidence in managing one’s own health and healthcare. Patients are categorized into 4 levels: Disengaged and overwhelmed, becoming aware but still struggling, taking action and gaining control, and maintaining behaviors and pushing further.^33^ DietID^™^ uses the patented Diet Quality Photo Navigation (DPQN^®^), which uses pattern recognition instead of dietary recall or food logs to provide a dietary assessment.^34^ Patients are presented composite images of established dietary patterns and are asked to select images most like their own, current food intake until a best fit is achieved. Outputs include the Healthy Eating Index (HEI-2020) and nutrient analysis. The ASA24 is a self-administered 24-hr recall dietary assessment developed by the National Cancer Institute.^35^

LFI: liver frailty index, LTPA: Godin-Shephard Leisure Time Physical Activity Index, PROMIS-29 v2.0: Patient Reported Outcomes Measurement Information System, DASI: Duke Activity Status Index, BIA: Bioimpedance analysis, ASA24: Automated self-administered 24-hour Dietary Assessment, PAM^®^: Patient Activation Measure

#### Body composition

Body composition will be measured using bioimpedance analysis (Quantum VII Biological Impedance Analyzer from RJL Systems) including fat, fat-free mass, lean dry mass, total body water, intra-cellular water, bone mineral content, lean soft tissue, skeletal muscle mass, phase angle, fat mass index, and fat free mass index. Anthropometric measurements including waist circumference, hip circumference, waist to hip ratio, waist to height ratio, midarm muscle circumference, and triceps skin fold will also be measured.

#### Laboratory data

Laboratory results to measure safety will be conducted at baseline, 6 weeks in the study, and 12 weeks in the study. Laboratory values to be obtained include liver function tests (AST, ALT, ALP, Total bilirubin) and kidney function (creatinine, eGFR, BUN, cystatin C).

### STRIVE Digital Health Program

STRIVE is a semi-supervised nutrition and exercise program delivered through the Heal-Me digital health application. Designed to improve frailty among older adults with cirrhosis, the program is structured into three pillars: Exercise, Nutrition, and Life Skills. The 12-week program includes home-based pre-recorded programs to be completed three times per week for 30 minutes. The mild-to-moderate intensity exercise program consists of functional, resistance, 30-second to 2-minute aerobic intervals, and stretch training. Patients are provided Therabands of varying difficulty to facilitate participation in home exercise videos. The nutrition and life skills pillars include videos, reading materials, interactive surveys, and life skills videos and surveys including developing readiness for change, SMART goals, self-compassion, sleep habits, and managing habits. Patients also have regular weekly check-ins with the clinical research coordinator, exercise specialist or physician and biweekly check-ins with a nutritionist to optimize patient’s engagement in study and provide personalized recommendations.

### Creatine/whey protein and placebo supplementation

Creatinine/whey protein and non-sugar based placebo powder supplementation will be provided at a dosage of 5g creatine and 35g whey protein daily or one daily serving of placebo powder respectively given 30 minutes prior to exercise on exercise days and anytime on days without exercise.^36^ This dose was determined based on general doses of creatine and/or whey protein supplementation provided in other studies among athletes and older adults.^27, 37^ Kidney and liver function will be evaluated at baseline, 6 weeks, and 12 weeks. Participants who develop a ≥ 5 times increase from baseline or upper limit of normal of AST/ALT, ≥ 1.5 times increase of creatinine, or any grade 3–4 laboratory abnormality will be evaluated for dose reduction or discontinuation. If patients experience moderate gastrointestinal symptoms interfering with daily activities, persistent muscle cramps, or ≥ 5 pound weight gain over 1 week with concern for fluid retention, participants will initially undergo reduction of supplementation to half dose at week 1 week and re-escalation to full dose at week 2 if symptoms resolve. If symptoms persist at half dose, supplementation is discontinued and patients will continue STRIVE digital health programming.

### Study Outcomes

The primary outcome will be physical frailty using LFI, a validated, cirrhosis-specific frailty measure consisting of grip strength, chair stands, and balance testing.^38^ We have previously found that a change in LFI of 0.3 units is associated with improved survival.^39^ We therefore define a change in LFI of at least 0.3 units during the course of the 12-week program as clinically significant in this study. Secondary outcomes will include change in HrQoL by PROMIS-29 v2.0, physical activity by DASI, healthy eating index by dietary pattern, and body composition by BIA and morphologic measures.

### Sample size

We aim to randomize 200 total participants (100 per arm) for this study. Calculations were performed using PASS sample size software and were based on two-sample t-test assuming equal variances with a significance-level of α = 0.05, testing the difference between two means, e.g., change in LFI between start of study and study completion between participants randomized to creatine/whey protein supplementation vs. placebo. Based on published cohorts that report a mean LFI of 3.8 (SD 0.72–0.77) in similar populations,^40^ the common standard deviation for both groups is assumed to be 0.75. The proposed sample size is powered at 80% to detect a mean change in LFI in the creatine/whey supplementation group that is 0.3 LFI-units greater than the mean change in LFI in the placebo group. We have previously found that a change in LFI of 0.3 units is associated with improved survival.^39^

### Data collection, validation, and management

Data will be collected and inputted into REDCap database. A clinical research coordinator will control data input before locking files within this study.

### Safety Management

An independent Data and Safety Monitoring Board will monitor interim data as the study progresses to ensure participant safety and review efficacy. The board will include two transplant hepatologists independent of the study and will review data, evaluate emerging safety information, adjudicate adverse event relatedness, patient discontinuation decisions, and other issues. Serious adverse events will be reviews monthly by the board.

### Statistical analysis

Analyses will be evaluated in the intention-to-treat population (all randomized participants). For the primary analysis, change in frailty (LFI) between the beginning of the study and study completion will be calculated for all participants, and the mean change will be modeled using a linear regression model that includes terms for trial arm (creatine/whey protein supplementation vs. placebo) and variables used in the randomization procedure (Child Pugh score and gender).^41, 42^Continuous secondary outcomes will be analyzed in the same way. In addition, changes in frail status (LFI ≥ 4.4) between the beginning of the study and study completion will be modeled using a logistic mixed effects regression model that includes fixed effects for trial arm and variables used in the randomization procedure and random effects for each participant to account for the within-participant correlation.

Exploratory models may adjust for prognostic variables and/or baseline variables that exhibit imbalance due to chance[cite]. Additional as-treated or per protocol analyses may be performed.

## Discussion

Older adults with cirrhosis represent a population of patients highly vulnerable to both age- and cirrhosis-related causes of frailty. Additionally, frailty is associated with accelerated clinical decompensation and death. Frailty therefore represents an attractive target to improve clinical outcomes, HrQoL, and healthcare expenditure.^8^

Chronic liver disease creates a catabolic state that drives the development of muscle dysfunction among adults with cirrhosis.^8^ Exercise and mobile-assisted exercise strategies have shown improvements in muscle health among adults with cirrhosis. A feasibility study of exercise and nutrition programming delivered on an earlier version of the Heal-Me digital application showed 70% engagement rates with an 89% satisfaction rate among 23 adults with cirrhosis with Child Pugh Class A and B cirrhosis.^43, 44^ Other home-based exercise programming among adults with cirrhosis have shown improvements in muscle-related factors including sarcopenia and physical frailty.^19^

Protein-energy malnutrition due to impaired nutrient intake, altered metabolism, and chronic inflammation, is another important pathway leading to frailty and sarcopenia among adults with cirrhosis.^8^ Nutrition prehabilitation with increased protein intake or supplementation have similarly shown improvements in frailty among adults with cirrhosis. In a randomized control trial of adults with compensated cirrhosis, 54 patients were enrolled to receive 16-week supplementation with branched-chain amino acids vs placebo twice daily. Patients on BCAA supplementation showed higher rates of frailty reversal (36% vs 0%, p < 0.001), significantly increased skeletal muscle index by BIA (7.5 vs 7.8, p = 0.03), and improved quality of life.^28^

These studies have contributed to national recommendations by the American Association of the Study of Liver Disease to prescribe exercise and nutrition counseling among adults with cirrhosis who are frail.^8^ However, these frailty-mitigating strategies often require in-person participation that leads to major barriers in access and provider resources, limiting adherence and scalability. Within the geriatrics literature, multimodal interventions and digital health formats have shown promising effects on frailty among older community-dwelling adults.^2, 20^ Multimodal interventions combining exercise and protein supplementation have synergistically improved sarcopenia and frailty among community-dwelling older adults. A randomized parallel group placebo controlled exploratory trial of physically inactive men and women ≥ 65 years showed the combination of supervised exercise training and whey and creatine supplementation showed improved hand grip strength, timed up and go, and timed chair stands compared to whey protein alone.^23^ A double-blind randomized placebo-controlled study of 48–72 year old men showed resistance training with placebo vs creatine vs whey protein vs creatine/whey protein supplementation showed improvements in muscle strength and mass among all groups with added supplementation compared to placebo alone.^26^ These and similar studies suggest that combination creatine/whey protein supplementation can be administered safely among community-dwelling older adults and may combat multiple etiologies of frailty within this population.

No prior studies have examined the effects of creatine/whey protein supplementation on frailty in older adults with cirrhosis, including those with decompensated cirrhosis. Therefore, this study aims to assess the effect of creatine/whey protein supplementation vs placebo with a digital health app providing exercise, nutrition, and life skills guidance on frailty among older adults with cirrhosis (Child Pugh Class A, B, and C). The findings from this study will guide recommendations for frailty-mitigating strategies among older adults with cirrhosis that may ultimately improve patient HrQoL hepatic decompensation, healthcare utilization and mortality.

## Abbreviations

STRIVE: Strength Training Intervention
PROMIS-29 v 2.0: Patient Reported Outcomes Measurement Information System
DASI: Duke Activity Status Index
PAM^®^: Patient Activation Measure
ASA24: Automated Self-Administered Dietary Assessment Tool (ASA24)
BIA: bioelectrical impedance analysis
LFI: liver frailty index
HrQoL: health-related quality of life
ATP: adenosine triphosphate
DPQN^®^: Diet Quality Photo Navigation
HEI-2020: the Healthy Eating Index
PAR-Q+: Physical Activity Readiness Questionnaire for Everyone
LTPA: Godin-Shephard Leisure Time Physical Activity Index
